# Identifying Consumers Who Search for Long-Term Care on the Web: Latent Class Analysis

**DOI:** 10.2196/10763

**Published:** 2018-11-02

**Authors:** Darren Liu, Takashi Yamashita, Betty Burston

**Affiliations:** 1 Department of Public Health College of Health Sciences Des Moines University Des Moines, IA United States; 2 Department of Sociology, Anthropology, and Health Administration and Policy College of Arts, Humanities, and Social Sciences University of Maryland, Baltimore County Baltimore, MD United States; 3 Department of Health Care Administration and Policy School of Community Health Sciences University of Nevada, Las Vegas Las Vegas, NV United States

**Keywords:** internet, information seeking behavior, consumer health information, marketing of health services, public reporting

## Abstract

**Background:**

Because the internet has become a primary means of communication in the long-term care (LTC) and health care industry, an elevated understanding of market segmentation among LTC consumers is an indispensable step to responding to the informational needs of consumers.

**Objective:**

This exploratory study was designed to identify underlying market segments of the LTC consumers who seek Web-based information.

**Methods:**

Data on US adult internet users (n=2018) were derived from 2010 Pew Internet and America Life Project. Latent class analysis was employed to identify underlying market segments of LTC Web-based information seekers.

**Results:**

Web-based LTC information seekers were classified into the following 2 subgroups: heavy and light Web-based information seekers. Overall, 1 in 4 heavy Web-based information seekers used the internet for LTC information, whereas only 2% of the light information seekers did so. The heavy information seekers were also significantly more likely than light users to search the internet for all other health information, such as a specific disease and treatment and medical facilities. The heavy Web-based information seekers were more likely to be younger, female, highly educated, chronic disease patients, caregivers, and frequent internet users in general than the light Web-based information seekers.

**Conclusions:**

To effectively communicate with their consumers, providers who target Web-based LTC information seekers can more carefully align their informational offerings with the specific needs of each subsegment of LTC markets.

## Introduction

### Background

It is widely acknowledged that the internet has become a primary marketplace for virtually all industries. Accordingly, potential consumers are now able to access necessary information for their decision making [1]. Today, health care and long-term care (LTC) providers, as well as the federal government, are also heavily reliant upon the internet to provide information [2,3]. Accordingly, it is unsurprising that consumers also rely on the internet to inform their buying decisions. Likewise, providers can now acquire a wealth of data via the Web to guide their marketing efforts.

Current research on Web-based information and knowledge exchange in the LTC marketplace reveals the criticality of these processes. For example, the 5-star rating system of the US Centers for Medicare and Medicaid Services (CMS) as a Web-based information exchange has significantly impacted the way LTC information is presented to the public and the way individuals perceive such information [4]. Introduced as Nursing Home Compare, a US public reporting system in December 2008, the findings from these report cards were first made available via the internet. These researchers used a before-and-after design to determine whether published data on quality measures of nursing homes created a shift in demand or in quantity demanded across nursing homes once consumers had more access to relevant information. Their study revealed that consumers of nursing home services decreased their purchase of institutional care from poorly rated facilities and increased the share of services bought from highly rated (ie, 5-star) facilities. Accessing such nursing home quality data via the internet was the primary vehicle that altered this important retail market adjustment process.

Additional research indicates that LTC providers also have an opportunity to promote their facilities by being responsive to the informational needs of consumers in areas that extend beyond the mainstream quality measures such as the Nursing Home Compare of the CMS. On the basis of focus groups and key informant interviews with persons aged 65 years and older, as well as family members of nursing home residents, a study found that there is a far greater breadth of informational needs than that which now exists on Nursing Home Compare [5]. Moreover, the nature of informational needs of consumers differs across various demographic segments of the American population. Similarly, another study, in an analysis of the information-seeking nursing home behavior on Yahoo! Answers, identified a wide range of consumer-based informational needs and a market-based discordance between the informational needs of current or prospective consumers in the LTC market, and the information made available by the LTC providers [6]. Indeed, this study suggested that nursing home sites may also need to provide assurances of quality care to family members of potential LTC consumers.

In order for nursing homes and/or other LTC organizations to effectively communicate their informational contents to the prospective consumers who seek information via the internet, more refined marketing segmentation is needed. Research must move beyond mere demographic and/or socioeconomic data so that the psychographic, sociographic, and/or clinical informational needs of various subsets of consumers can be addressed. In this respect, an analytical approach that allows the identification of subgroup differences in the Web-based informational needs of consumers is useful [7].

The internet provides an opportunity for all marketplaces to function more optimally as a source of timely information. Yet, there is paucity of research that specifically examines how LTC information is accessed, and how the available Web-based information is in alignment with the information sought. This study seeks to initiate the process of remediating this void by advocating the use of market segmentation to better facilitate the exchange of Web-based information between LTC consumers and providers.

### Objectives

Building upon the insights from previous research that focused on health and medical information-seeking behaviors [8-10], this exploratory study uses a large dataset of American adults to segment internet users who seek Web-based LTC information. Moreover, this research profiles the Web-based information-seeking behaviors of identified subgroups of internet users. Specifically, this study uses latent class analysis (LCA)—a person-centered approach—to accomplish this task. A content analysis of past studies reveals a sole reliance on the adoption of variable-centered approaches, such as linear regression and logistic regression, to determine whether significant differences in information-seeking activities occurred. This is not to say that such approaches are methodologically flawed. Indeed, the opposite is true.

Variable-centered approaches are sound when examining relationships between variables and developing the initial segmentation basis for internet users. However, such methodologies also embody several limitations in circumstances when more detailed segmentation data are required. First, the estimation and interpretation of models with more than one outcome variable can be a challenging task. As such, variable-centered approaches are generally not suitable for the simultaneous examination of multiple internet users’ information-seeking behaviors (eg, the data we used for this study). Second, the extent to which such statistical models are capable of identifying the characteristics of target populations is also somewhat restricted. Specifically, the effect of one characteristic (eg, gender or education) on the outcome variable can only be examined while all other characteristics are held constant. Third, the traditionally used variable-centered approach measures an average effect of a predictor variable on the outcome variable by using the premise that all individuals were sampled from the same population. Such an approach explicitly bypasses underlying subpopulation differences. Finally, in conjunction with the first three limitations, variable-centered approaches do not clearly identify the consumer subpopulations to whom LTC providers must be responsive at a micro-level. Indeed, most studies on the information-seeking behavior of consumers fail to consider the need for providers to direct responsive *answers* to these information-seeking consumers so that overall health outcomes can improve.

This study used one of the first publicly available consumer survey datasets that include the questions of internet search for LTC. This study was specifically designed to address the limitations of the currently dominant variable-centered approaches while building upon the findings from previous studies on Web-based health or medical information-seeking. Moreover, this inquiry broadens dialogue by employing LCA [11]. LCA has been increasingly used in medical, health, social, and behavioral sciences [7]. The primary strength of this approach is the identification of underlying subpopulations that share similar sets of behaviors while separately developing profiles of multiple subpopulations. LCA assumes that unobserved groups (latent classes) are present and that these groups have highly refined needs and behaviors [11].Rather than modeling associations between variables, LCA first detects and then characterizes previously unobserved groups of persons (ie, subpopulations) within the larger sample.

The use of the person-centered approach supports the profiling of the internet users who seek LTC information while simultaneously taking other factors into account. These factors include (1) a summary description of the multiple health information-seeking behaviors displayed and (2) the construction of a sociodemographic profile of the internet users by identified subgroups. When LTC providers better understand the informational needs of each subgroup, they can better respond to these needs via their website and/or other marketing materials. This study was designed to answer the questions listed below:

1. Who are the subgroups or unique market segments that search the internet for LTC information?
2. What health, medical, and/or other knowledge is sought by the internet users who seek Web-based LTC information?
3. What are the sociodemographic and other characteristics of the internet users who search the internet for LTC information?

Based upon the answers to the above questions, recommendations can be made to LTC information providers regarding the type of information they should disseminate via Web-based resources.

## Methods

### Data Sources

Data from the 2010 Princeton Survey Research Associates International for the Pew Internet and American Life Project (Pew Internet) were used to answer the 3 described questions [12]. Collected through telephone interviews with adults aged 18 years and older in August and September of 2010, the samples for this study were drawn from a pool of 20,985 landline users and 12,699 cell phone holders by Survey Sampling International, LLC [13]. The Pew Internet database explores the impact of the internet on children, families, communities, the work place, schools, health care, and civic or political life. In 2010, the Pew Internet and American Life Project included, for the first time ever, a question regarding LTC health information-seeking over the internet. The original survey item asked the respondents whether they had searched the Web for information about a series of health and medical topics. LTC was one of the response categories.

A series of survey items were included to assess key sociodemographic characteristics, internet use behaviors, and the health-related information sought by seekers of LTC information. Random digit dialing was used as a sampling strategy. Although not entirely representative of all of the US adult population, the data covered a large population of phone users [14]. Because this unique dataset collects Web-based health, medical, and LTC information-seeking behaviors, it provides a unique opportunity for researchers to conduct a market segmentation study based on internet use for LTC information. After excluding noninternet users (n=976) and missing values for key Web-based information-seeking behaviors (n=7) from the original sample (n=3001), the final sample size was 2018.

### Measures

#### Outcome Variable

**Figure 1:**
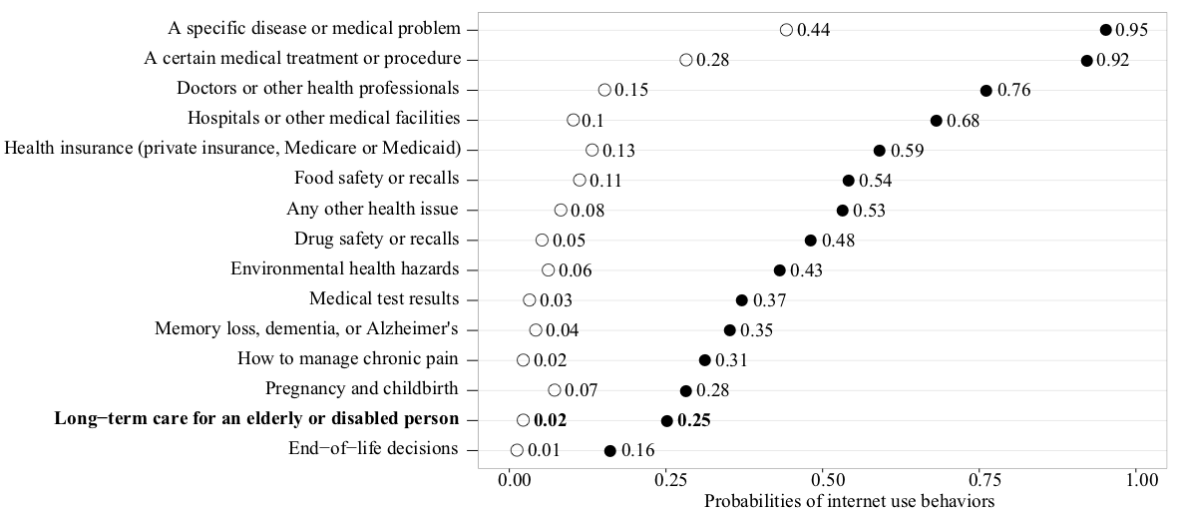
Posterior probabilities for the online information-seeking behaviors by the identified latent classes.

The primary outcome of interest was a dichotomous measure indicating 2 identified latent classes (which is labeled as class 1 [light information seekers] vs class 2 [heavy information seekers, reference group]). Using LCA (described more in the next sections), these subgroups were identified based on a set of 15 Web-based health information-seeking behaviors with dichotomous responses (Yes or No; see Figure 1). The health information-seeking referred to looking for both the long-term information and other health-related information for themselves or for someone else.

#### Predictor Variables

A variety of demographic, socioeconomic, health status, and caregiving status information was included for each model. Age was recorded in years. However, people older than 97 years were top-coded at 97. The more traditional demographic and socioeconomic segmentation variables were included as predictor variables. These included (1) gender (women vs men); (2) race or ethnicity (black vs white, Hispanic vs white, and others vs white); (3) marital status (married vs not married); and (4) employment status (employed vs not employed and retired vs not employed). These dichotomous measures were used for purposes of cross-classification. The number of people in each household was measured as the absolute count of total household members. Educational attainment was assessed based on a 5-point Likert-scale (1-5: None-Postgraduate degree). Household income was recorded using a 9-point Likert-scale ranging from less than US $10,000 to US $150,000 or more. Uneven rather than even increments were used. As a result, the income classes could not be treated as a continuous variable (eg, by US $10,000, US $25,000, and US $50,000). Self-rated health was recorded based on a 4-point Likert-scale (1-4: Poor-Excellent). However, a range of clinical variables, as well as other segmentation factors were included. The number of self-reported chronic conditions was counted based on following 6 major diseases: diabetes mellitus, hypertension, lung disease, cardiovascular disease, cancer, and/or other chronic diseases. Disabilities were accessed using 6 disability indicators based on difficulties with hearing, vision, memory, walking, dressing, and running errands. Two dichotomous measures of caregivers were used as follows: (1) caregivers for 1 or more parents vs noncaregivers; (2) caregivers for adults who were not parent(s) vs noncaregivers (reference group). Finally, internet usage was recorded based on 7-point Likert-scale (1-7: Never-Several times a day) either at home or at work. Internet users who reported “Never” but still used email were classified as internet users in this study. Accordingly, the study included the potentiality for many subsegments based on various permutations and combinations of the included categories.

#### Analytic Strategies

**Figure 2:**
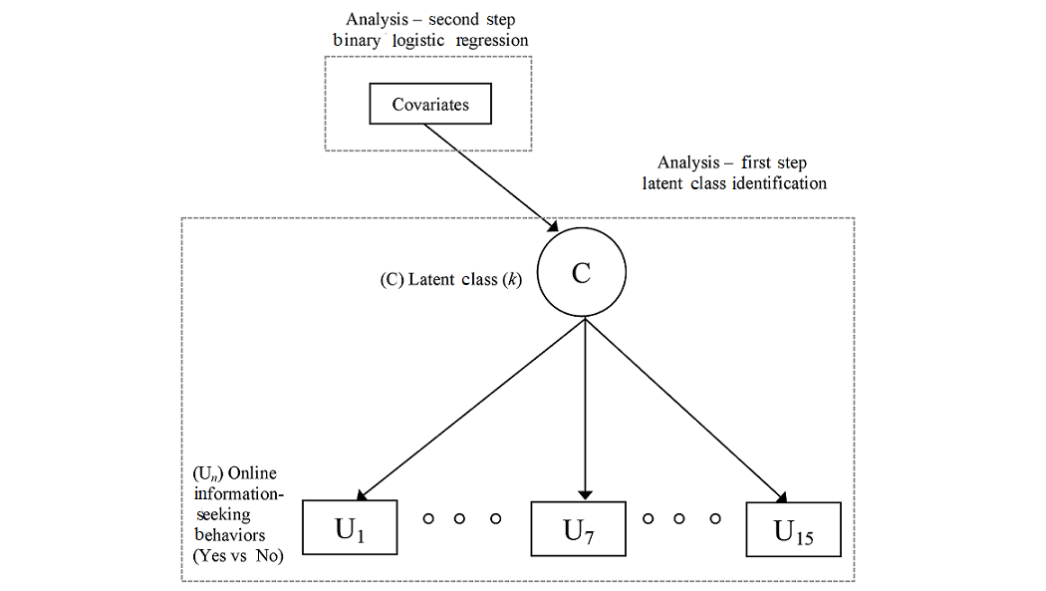
Latent class analysis model and analytic approaches.

Two primary areas of inquiry guided this research. At the first level, this study sought to identify unique underlying subgroups or market subsegments that used the internet to address their needs relative to informational LTC. This study also sought to identify the health- or medical information-seeking activities of consumers across various subgroups. Accordingly, the first part of the analysis focused on the identification of the latent classes of users. Figure 2 presents a path diagram of the theoretical proposition that was applied for latent class analysis. The analysis was completed in 2 sequential steps using Mplus version 7 (Muthén and Muthén).

First, an LCA was conducted using the 15-Web-based health-related and medical information-seeking behaviors. LCA is a special type of structural equation model (SEM) with unobserved or latent variable(s) [11]. Latent variables are commonly modeled with continuous observed variables (eg, measurement model) [15]. In other words, LCA is an SEM with a categorical latent variable [16]. The number of final groups was chosen based on the average posterior class membership probability, classification quality, and interpretability in view of possible implications of the findings for LTC providers relative to the type of information they should supply via Web-based mechanisms [7]. In the preliminary analysis, the number of groups (*k*) was set between 2 and 6 in LCA, and full information maximum likelihood estimation was applied. With each *applicant*, several variables were analyzed, including (1) the specific group membership probability (0.7 or higher) [17]; (2) the percentage of people in the smallest class; (3) the classification quality indicator [11] (entropy>0.8); (4) bootstrap likelihood ratio test [18] (BLRT: *k* vs *k* −1 specification); and (5) Akaike information criteria (AIC). Bayesian information criteria (BIC) and interpretability [19] were also evaluated (see Table 1).

As a result, the model with 2 latent classes was determined to be optimal (the posterior membership probabilities>0.95; entropy=0.84, and BLRT *P*<.05). Although AIC and BIC were smaller as the number of classes increased, other criteria indicated (eg, entropy) that the model with 2 or 3 classes was *finer*. However, the 2-class specification was chosen in view of the interpretability [19]. On a related note, the covariates were not included in the final LCA model because of unstable identification of the latent classes. However, given the high-quality classification [20] (entropy greater than 0.8) and the purpose of this study (ie, profiling) or segmentation, the effects of covariates on each class membership were examined in the second step of the analysis.

### Two Latent Subgroups

Each latent class corresponded with an underlying subgroup of internet users who visit the Web in search of information about the LTC marketplace. Figure 1 describes the percentages of internet information-seeking behaviors by the 2 classes. As can be seen, the class 1 members (black dots) are appreciably more likely to seek health, medical, and LTC information than the class 2 members (white dots). Moreover, for each specific Web-based information-seeking behavior, the pattern was consistent (ie, the class 1 is higher than the class 2).

In terms of LTC Web-based information-seeking, the difference between these 2 classes was evident. The first latent class is characterized by a high probability of internet use behavior. As a result, class 1 users were labeled as *heavy Web-based information seekers*. In contrast, class 2 is characterized by a low probability of internet use behavior. Thus, this segment of LTC current or prospective consumers was labeled as *light Web-based information seekers*.

**Table 1:** Comparisons between the latent class analyses with different number of latent classes.

Model selection criteria	*k*^a^=2	*k*=3	*k*=4	*k*=5	*k*=6
The minimum percentage of 1 class	44.50	22.8	15.46	10.50	7.17
The mean posterior class membership probability^b^	>0.96	>0.91	>0.83	>0.78	>0.77
Entropy	0.84	0.82	0.78	0.77	0.79
Bootstrap likelihood ratio test (*k* vs *k*-1)-2 log likelihood (degrees of freedom)	5057.04 (16)^c^	1010.30 (16)^c^	274.03 (16)^c^	182.95 (16)^c^	141.77 (16)^c^
Akaike information criteria	27,465.22	26,486.93	26,244.90	26,093.95	25,984.18
Bayesian information criteria	27,639.12	26,750.59	26,598.32	26,537.13	26,517.12

^a^*k*: number of latent classes.

^b^The model with *k*=2 was selected as the final model considering the highest posterior class membership probability, entropy, statistically significant difference from the model with k=3, and interpretability (ie, more distinctive internet use behaviors between classes).

^c^*P*<.001.

Table 2 represents a descriptive summary of both classes of users. The proportional odds binary logistic regression [21] was used to examine the effects of both sociodemographic and other market segmentation variables. Specifically, the impact of health status, caregiving status, and internet usage on membership of each primary class (ie, heavy vs light Web-based information seekers) was evaluated. It is important to note that SAS version 9.4 (SAS Institute Inc, Cary, NC, USA) was used because Mplus version 7 does not return the c-statistic [21,22] that was used to assess model quality. This fact assumes importance, given that SAS and Mplus, at the time of this study, do not use the identical estimation algorithms. Therefore, the computed c-statistic may require caution in its interpretation.

**Table 2:** Descriptive summary of internet users by the identified latent classes.

Characteristics		Latent class 1; heavy Web-based information seekers (n=1120)	Latent class 2; light Web-based information seekers (n=898)
Age in years, mean (SD)		44.30 (15.97)	45.03 (17.91)
**Gender, n (%)**			
	Women^a^	713 (63.70)	479 (53.3)
**Race or ethnicity, n (%)**			
	White^b^	754 (67.31)	547 (60.9)
	Black^c^	173 (15.43)	176 (19.6)
	Latino	132 (11.77)	122 (13.6)
	Others	61 (5.49)	53 (5.9)
Married (vs not married)^c^, n (%)		604 (53.91)	417 (46.4)
Number of household members, mean (SD)		2.18 (0.92)	2.19 (0.98)
**Educational attainment^a^, n (%)**			
	High school or less	41 (3.62)	84 (9.3)
	Vocational school	246 (22.00)	317 (35.3)
	Some college or associated degree	24 (2.17)	25 (2.8)
	Bachelor’s degree	405 (36.18)	279 (31.0)
	Postgraduate degree	404 (36.03)	194 (21.6)
**Employment status, n (%)**			
	Employed^b^	753 (67.19)	541 (60.2)
**Annual household income in US $^a^, n (%)**			
	Less than $10,000	50 (4.45)	93 (10.3)
	$10,000 to under $20,000	104 (9.28)	90 (10.0)
	$20,000 to under $30,000	97 (8.64)	107 (12.0)
	$30,000 to under $40,000	131 (11.69)	120 (13.4)
	$40,000 to under $50,000	104 (9.28)	110 (12.3)
	$50,000 to under $75,000	206 (18.42)	152 (16.9)
	$75,000 to under $100,000	168 (14.99)	106 (11.9)
	$100,000 to under $150,000	144 (12.83)	69 (7.7)
	$150,000 or more	117 (10.42)	52 (5.6)
Insured^a^, n (%)		1009 (90.12)	760 (84.6)
**Self-rated health, n (%)**			
	Excellent	365 (32.55)	299 (33.3)
	Good	609 (54.40)	475 (53.0)
	Only fair	126 (11.27)	105 (11.7)
	Poor	20 (1.78)	18 (2.1)
Number of chronic conditions^c^, mean (SD)		0.21 (0.50)	0.63 (0.93)
Number of disabilities, mean (SD)		0.33 (0.75)	0.33 (0.75)
**Caregivers for adults**			
	Parents vs noncaregivers^a^, n (%)	178 (15.91)	77 (8.5)
	Nonparents vs noncaregivers^a^, n (%)	250 (22.31)	127 (14.1)
**Internet usage^a^, n (%)**			
	Never^d^	22 (2.00)	31 (3.4)
	Less often	11 (1.00)	35 (3.9)
	Every few weeks	15 (1.34)	39 (4.4)
	1-2 days a week	55 (4.90)	92 (10.3)
	3-5 days a week	110 (9.80)	125 (14.0)
	About once a day	147 (13.14)	177 (19.7)
	Several times a day	760 (67.82)	398 (44.4)

^a^*P*<.001 for the *t* test or chi-square test.

^b^*P*<.01 for the *t* test or chi-square test.

^c^*P*<.05 for the *t* test or chi-square test.

^d^Internet usage: never, these respondents still used email and, therefore, classified as internet users.

## Results

### Findings From the Analysis of Posterior Probabilities

The findings from the analysis reveal a contrariety. Although a priori reasoning would suggest that people in need of LTC and/or individuals with a chronic disease would be more compelled to use the internet for information-seeking, this was not the case. As mentioned, 2 latent classes existed among the study participants: heavy Web-based information seekers (n=1120) and light Web-based information seekers (n=898). The heavy Web-based information seekers were more likely to be women (independent of race or ethnicity). These women were most often married, highly educated, employed, economically upper class, insured, less chronically ill, and in general, more active internet users.

Unsurprisingly, the heavy Web-based information seekers (15.91%, 178/1120) were more likely to be caregivers than the light Web-based information seekers (8.5%, 77/898). In this study, about 25% of the heavy Web-based information seekers reportedly looked for LTC information on the Web, whereas only about 2% of light Web-based information seekers did so (see Figure 1). Moreover, the individuals who sought LTC information on the Web were also more likely to use the internet to look for other health and medical information. Specifically, majority of the heavy Web-based information seekers looked for health and medical information related to a specific disease, medical treatment, health care professionals, hospitals, insurance, food safety, and other health issues.

### Results of the Binary Logistic Regression

The results of the binary logistic regression were predictive of the latent class membership. This analysis revealed 8 statistically significant predictors (see Table 3). Interestingly, older adults were less likely to be the heavy Web-based information seekers and, therefore, less likely to seek Web-based LTC information, compared with younger adults. The membership of heavy Web-based information seekers was predicted by female gender, higher education, higher household income, and a greater number of chronic conditions. As expected, caregivers to parents and caregivers to adults who were not their parents had 1.94 times and 1.82 times odds of being the heavy Web-based information seekers than noncaregivers to any adult. That is, caregivers were significantly more likely to look for the LTC information, as well as other Web-based information than noncaregivers.

Finally, the adults who used the internet more often also tended to be in the category of heavy Web-based health-related as well as LTC information seekers. Overall, individuals who had health issues (either their own or someone else’s) and/or caregiving responsibilities and particular characteristics (eg, gender and higher socioeconomic status) were significantly more active in terms of Web-based health and medical and LTC information-seeking behaviors.

**Table 3:** Estimated odds ratios from proportional odds binary logistic regression on the heavy Web-based information seekers (class 1) versus light Web-based information seekers (class 2).

Variables	Odds ratio (SE)
Age (years)	0.98 (0.01)^a^
Women (vs men)	1.90 (0.13)^a^
Black (vs white)	0.77 (0.17)
Latino (vs white)	0.89 (0.19)
Others (vs white)	0.64 (0.29)
Married (vs not married)	1.17 (0.15)
Number of household members	0.94 (0.07)
Educational attainment	1.34 (0.05)^a^
Employed (vs not employed)	1.02 (0.17)
Retired (vs not employed)	1.27 (0.26)
Annual household income	1.09 (0.03)^b^
Insured (vs uninsured)	1.09 (0.19)
Self-rated health	0.96 (0.10)
Number of chronic conditions	1.30 (0.08)^a^
Number of disabilities	1.06 (0.09)
Caregivers for adults (parent vs noncaregivers)	1.94 (0.20)^a^
Caregivers for adults (nonparent vs noncaregivers)	1.82 (0.17)^a^
Internet usage	1.20 (0.05)^a^

^a^*P*<.001.

^b^*P*<.05.

## Discussion

### Principal Findings

This exploratory study analyzed a large dataset of the internet users and identified the primary segments of users, as well the subsets within each larger segment of the adults who looked on the Web for LTC and other health and medical information. However, the implications of this study extend far beyond the defined areas of inquiry. Although multiple sources of LTC information exist on the Web, LTC providers as the *suppliers* in the markets can use findings from this study to ensure that the Web-based information that they provide is easily accessible by these various segments of users and includes the types of information that these various segments seek. The following sections provide brief discussions on the selected areas for future research.

### Heavy Versus Light Web-Based Information Seekers

One important finding from this study is the unobserved latent class memberships among the heavy and light Web-based information seekers. The latent class membership is informative of Web-based LTC of multiple individuals and other health and medical information-seeking behaviors. That is, when individuals seek LTC information on Web, there is a significantly greater chance that they also use the internet to look for other health and medical information. Moreover, with the traditional variable-centered approach, findings are limited to the associations between two variables at a time while holding all other variables or covariates constant (ie, assuming all other variables are the same). Two practical implications can be drawn from the finding.

First, the volume of research on Web-based health and medical information is significantly greater than that of LTC information [23-26]. Accordingly, as LTC providers deliver knowledge and information to current and/or prospective users, this Web-based LTC information should be designed in alignment with the rich literature on Web-based health and medical information. Thus, this study provides a foundation for improvements in the *fit* between Web-based information-seeking by LTC consumers and the information made available by LTC providers. For example, given the heavy information seekers use the internet for multiple purposes, packaging the geriatric medical information (eg, aging-related disabilities and dementia) and associated LTC information in one place or website may help streamline efficient information-seeking experience.

Second, LTC providers, depending on the nature of their services, can literally target the specific subpopulations identified in this study. For example, if the goal is to provide Web-based LTC information to older adults who may need LTC services at some point in the future, LTC providers can target light Web-based information seekers who can be identified based on a set of characteristics, including age, gender, educational attainment, household income level, number of chronic conditions, caregiving responsibilities, and general internet use. In other words, given that most of the light information seekers do not use the internet for health, medical, and LTC information, more aggressive marketing and outreach with the traditional health communication (eg, printed materials such as flyer and postcard) may be necessary.

Similarly, given the findings on the heavy Web-based information seekers, LTC providers can align their messages with non-LTC health and medical information sources. This is a highly effective strategy for reaching their audiences given that Web-based information seekers tend to simultaneously look for LTC and health and medical information. Finally, more practical strategies can also be used to better coordinate LTC providers and consumers’ health and medical information as additional research of this type is completed.

### Younger Versus Older Adults

This study reconfirmed findings from other researchers, which indicated that older adults, despite their status as the primary LTC consumer segment, are significantly less likely to seek LTC information on the Web. Citing data from the Pew Internet Report, this research confirms that although internet use has been increasing among older adults, usage levels continue to remain below those of younger adults [27]. However, also using data from the Pew Research Center, another study found that in 2013, 53% of adults aged 65 years and older used the internet [28]. Yet, although 86% of this total communicated via email, a mere 27% used the internet for improving their health literacy through health-related information-seeking. The data suggest that there is an urgent need for LTC providers to assume a role of leadership in directing internet-based social marketing toward seniors. Munshi et al [29], describe the robust need for diabetes care among many LTC consumers. As this study reveals, health area–specific unique informational needs exist among LTC consumers with chronicities.

### Information Gaps Between Long-Term Care Consumers and Providers

A greater informational exchange between this subsegment of LTC consumers and providers can potentially improve outcomes via better informed decision making in LTC preplanning before the emergence of aging-related severe cognitive and/or physical disabilities that require LTC services. That is, a knowledge informational gap in the LTC marketplace that can only be addressed when providers and consumers of LTC experience better coordination in the online demand for, and supply of, LTC information [30]. As is known, the LTC marketplace as currently structured is one that is built upon minimum levels of dialogue between consumers and/or their representatives and LTC providers. This tendency is revealed as one reviews the US CMS document, *Your Guide to Choosing a Nursing Home or Other Long-Term Care* [31]. This document recommends the use of Eldercare Locator, Agency, and Disability Resource Centers (ADRCs), Long-Term Care Ombudsman, and other services. However, it also reveals the need for more direct informational linkages between LTC consumers and LTC providers. Again, an awareness of the unique informational needs of LTC subsegments can be used to improve this dialogue.

### Women Versus Men as Seekers of Long-Term Care Information on the Web

One study using data from 7609 Medicare beneficiaries in the 2011 National Health and Aging Trends Study, found that, in general, males are more likely to use the internet than females [27]. Yet, the results from this study revealed that females, perhaps because of their over-representation among caregivers, were more likely to seek Web-based information on LTC than males. This finding suggests that if the LTC industry wishes to direct internet messaging to these unique segments of information seekers, separate messaging content and information dissemination strategies will be required. A similar pattern of research-driven market segmentation can also be found in any industry [32].

### Access to the Internet: People With Higher Versus Lower Socioeconomic Status

This study, as has been true with other analyses, also discovered that people with higher incomes and higher levels of education are more likely to access LTC information on Web. Yet, in some respects, people having lower income with disabilities that require LTC find themselves engaged in a more complex network of financial transactions as they engage in eligibility screening (eg, Medicaid), benefits establishment, and dual-eligibility [33]. People with lower income because of lack of exposure to technology or financial resources [34] are disproportionately likely to rely on cell phones rather than personal computers [35]. LTC providers may consider disseminating information to this group via mobile phone apps and/or mobile-friendly websites.

### People With More Versus Fewer Chronic Diseases

The findings also reveal that persons with chronic diseases are more likely to engage in LTC and health-related information-seeking on the internet (arguably out of necessity). This finding on chronic conditions and Web-based information-seeking suggests that LTC providers can disseminate reliable information to prospective residents regarding their services for managing various chronicities. One study criticizes the internet as a source of health information based upon fragilities, complexity of the information, and the observed frequency of inaccurate information [36]. Accordingly, LTC providers will need to ensure that the targeted market subsegments are delivered accurate information in a format compatible with their informational needs [37].

### Conclusions

This exploratory study applied LCA as a tool for the segmentation of LTC internet information-seeking into relevant subsegments. Such a person-centered approach can potentially improve the operations of LTC marketplace. The analysis of a large pool of data of American adults identified two underlying market segments—heavy and light Web-based information seekers—according to their Web-based LTC and health and medical information-seeking behaviors. The study also revealed that the segmentation basis for LTC consumers includes but extends beyond demographic and socioeconomic variables such as age, gender, educational attainment, and household income level. Rather, chronic conditions, caregiving status, and general internet usage are predictive of class membership. Thus, identifying the latent classes with more or less usage of the internet for LTC and health and medical information was merely a starting point. The next step involves using the findings from this study to enhance Web-based communications between LTC providers and current and prospective LTC consumers. In this respect, this exploratory analysis contributed to the framework for future research and provided a foundation to generate greater dialogue regarding how various subsegments of LTC information seekers via the internet can be better linked with LTC service providers, the group that is best positioned to deliver information essential for decision-making.

## Abbreviations

AIC: Akaike information criteria
BIC: Bayesian information criteria
BLRT: bootstrap likelihood ratio test
CMS: Centers for Medicare and Medicaid Services
LCA: latent class analysis
LTC: long-term care
SEM: structural equation model
